# Reproducible Colonization of Germ-Free Mice With the Oligo-Mouse-Microbiota in Different Animal Facilities

**DOI:** 10.3389/fmicb.2019.02999

**Published:** 2020-01-10

**Authors:** Claudia Eberl, Diana Ring, Philipp C. Münch, Markus Beutler, Marijana Basic, Emma Caroline Slack, Martin Schwarzer, Dagmar Srutkova, Anna Lange, Julia S. Frick, André Bleich, Bärbel Stecher

**Affiliations:** ^1^Max von Pettenkofer-Institute, LMU Munich, Munich, Germany; ^2^German Center for Infection Research (DZIF), LMU Munich, Munich, Germany; ^3^Department for Computational Biology of Infection Research, Helmholtz Center for Infection Research, Brunswick, Germany; ^4^Institute for Laboratory Animal Science and Central Animal Facility, Hannover Medical School, Hanover, Germany; ^5^Institute of Food, Nutrition and Health, ETH Zürich, Zurich, Switzerland; ^6^Institute of Microbiology of the Czech Academy of Sciences, Nový Hrádek, Czechia; ^7^Institute of Medical Microbiology and Hygiene, University of Tübingen, Tübingen, Germany; ^8^German Center for Infection Research (DZIF), Tübingen, Germany

**Keywords:** syncom, Oligo-MM12, sDMDMm2, minimal microbiome, 3R, gnotobiology, defined bacterial consortia, isobiotic mice

## Abstract

The Oligo-Mouse-Microbiota (OMM^12^) is a recently developed synthetic bacterial community for functional microbiome research in mouse models (Brugiroux et al., 2016). To date, the OMM^12^ model has been established in several germ-free mouse facilities world-wide and is employed to address a growing variety of research questions related to infection biology, mucosal immunology, microbial ecology and host-microbiome metabolic cross-talk. The OMM^12^ consists of 12 sequenced and publically available strains isolated from mice, representing five bacterial phyla that are naturally abundant in the murine gastrointestinal tract (Lagkouvardos et al., 2016). Under germ-free conditions, the OMM^12^ colonizes mice stably over multiple generations. Here, we investigated whether stably colonized OMM^12^ mouse lines could be reproducibly established in different animal facilities. Germ-free C57Bl/6J mice were inoculated with a frozen mixture of the OMM^12^ strains. Within 2 weeks after application, the OMM^12^ community reached the same stable composition in all facilities, as determined by fecal microbiome analysis. We show that a second application of the OMM^12^ strains after 72 h leads to a more stable community composition than a single application. The availability of such protocols for reliable *de novo* generation of gnotobiotic rodents will certainly contribute to increasing experimental reproducibility in biomedical research.

## Introduction

The mammalian gut is a complex ecosystem, hosting a diverse microbial community that influences normal physiology and disease susceptibility (mainly) through its metabolic activities. The gut microbiome is highly dynamic throughout life and can vary substantially between individuals due to diet, lifestyle and genetic factors (Ursell et al., 2012; Falony et al., 2016). Microbiome community profiling and metagenome-based approaches have recently elucidated how inter-individual microbiome differences correlate with disease states and health (Knight et al., 2017; Hadrich, 2018). However, causal relationships between the microbiota and disease conditions can rarely be deciphered with these methods. Ethical and regulatory issues further limit the possibilities of human intervention studies and robust experimental animal models are urgently needed to research the causality between the gut microbiota and various human diseases.

The laboratory mouse is currently the primary experimental model organism in biomedical research (Taylor et al., 2008). The availability of numerous genetically engineered and mutant mouse strains greatly facilitates functional studies (Eppig et al., 2015). Mice raised in different research institutions, obtained from different vendors or the wild can exhibit profound differences in microbiota composition (Stecher et al., 2010; Wang et al., 2014; Rausch et al., 2016; Sadler et al., 2017; Thiemann et al., 2017), mimicking the inter-personal microbiota variation in human populations. Apart from vendor-specific microbiomes, genotype and environmental conditions such as diet, cage-type, temperature and bedding can profoundly influence microbiota composition and function (Hildebrand et al., 2013). These microbiome differences between genetically identical mouse models have led to the serendipitous discovery of numerous microbiota-dependent disease phenotypes in the past, including immune-cell priming, colitis susceptibility and resistance to infections (Ivanov et al., 2009; Surana and Kasper, 2017; Velazquez et al., 2019). Overall, there is growing evidence that the microbiota is a major confounding factor, which complicates cross-study comparisons and eventually jeopardizes experimental reproducibility (Franklin and Ericsson, 2017). To identify the mechanisms of host genetics-imposed control of the microbiome, adequate experimental design is instrumental (Laukens et al., 2015; McCoy et al., 2017; Mamantopoulos et al., 2018). In this respect, gnotobiology (greek: gnosis: knowledge; bios: life; logos: study) has gained importance as it allows for optimal control of microbiota within and in between animal facilities. Besides, gnotobiology has become an essential method to mechanistically investigate microbiota functioning and to assess causality in disease-associated alterations of gut microbiota composition (Trexler and Reynolds, 1957; Orcutt et al., 1987). Gnotobiotic mice can be generated by microbial reconstitution of germ-free mice with fecal transplants, single organisms and defined mouse- or human-derived microbial consortia (Clavel et al., 2016; McCoy et al., 2017). Several recent studies have focused on the isolation of bacteria from humans and mice and the establishment of culture collections (Hugon et al., 2015; Lagkouvardos et al., 2016; Forster et al., 2019). The most important requirement for the assembly of defined bacterial consortia is the availability of well-characterized and genome-sequenced strains – preferably in public culture collections. Further, state of the art methods to trace and quantify each member of a microbial consortium are needed for quality control and functional studies. Finally, experimental protocols should be optimized to ensure reproducible colonization of germ-free mice with a given microbial consortium.

A majority of studies uses human-derived bacteria to colonize gnotobiotic mice even though phylogenetic differences between human and mouse microbiota (Xiao et al., 2015) may affect microbe-host interaction and long-term stability of colonization. To circumvent this problem, we have developed a model based on twelve bacteria from the murine gut microbiota (Brugiroux et al., 2016; Garzetti et al., 2017). This Oligo-Mouse-Microbiota (OMM^12^) provides several improvements to other defined consortia, including broad phylogenetic diversity, public availability of the strains from the German Type Culture Collection^1^ (Brugiroux et al., 2016). Most importantly, the OMM^12^ exhibits long-term stability in gnotobiotic mice (Brugiroux et al., 2016). Correspondingly, C57BL/6 mice stably colonized with OMM^12^ have recently also been designated stable defined moderately diverse microbiota mice (sDMDMm2) (Li et al., 2015). The Altered Schaedler Flora (ASF), another frequently used model for gnotobiotic research for more than 40 years is also based on murine bacteria (Wymore Brand et al., 2015). Compared to ASF-colonized mice, OMM^12^ mice show increased resistance to pathogen colonization (Brugiroux et al., 2016). These characteristics indicate that OMM^12^ mice mimic the normal physiology better than ASF mice and are therefore preferable for microbiome intervention studies that investigate microbiome-correlated diseases. Accordingly, several studies have already implemented the OMM^12^ as model to systematically probe for a causal role of individual microbes in protection against different pathogens along the lines of Koch’s postulates (Brugiroux et al., 2016; Studer et al., 2016; Herp et al., 2019). To date, the OMM^12^ model is used by over 30 research groups world-wide to address fundamental research questions related to microbial ecology, metabolism, mucosal immunology and infection biology (Li et al., 2015; Studer et al., 2016; Uchimura et al., 2018).

Here, we present a feasibility trial and scrutinize colonization of germ-free C57BL/6 mice with the OMM^12^ community. We established a protocol using a standardized OMM^12^ inoculum and compared community composition when inoculations were performed repeatedly in the same facility or at different facilities. We show that double inoculation leads to efficient introduction of consortium members in different facilities. Notably, we observed subtle differences in the absolute abundance of some of the strains between different experiments. This also translates to differences in community profiles. Whether this induces functional changes to the community and phenotypic differences in the mice should be a matter of future investigation. Still, care should be taken when comparing results obtained with the model at different facilities. Overall, our study shows that generating mice colonized with the OMM^12^ synthetic bacterial consortium shows excellent reproducibility between different animal facilities.

## Materials and Methods

### Animal Facilities

**FIGURE 1:**
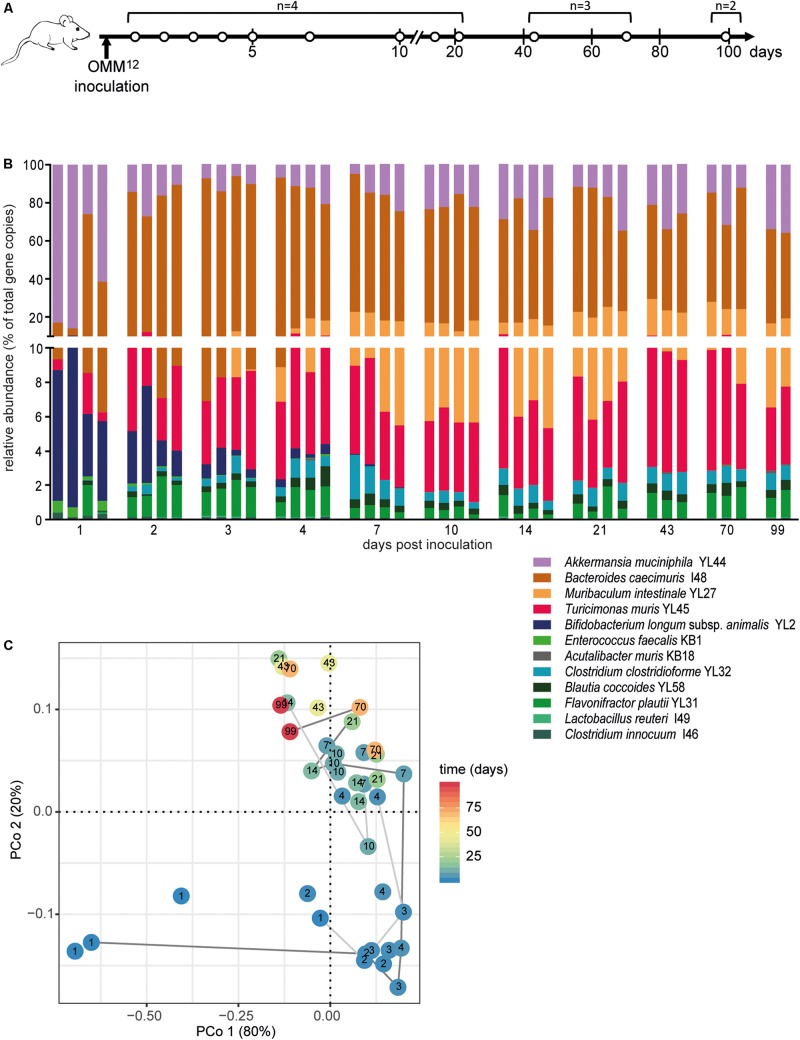
Dynamics of fecal community composition after inoculation of OMM^12^ colonization in germ-free mice. **(A)** Experimental scheme. Germ-free mice were inoculated with the OMM^12^ mixture and kept in a germ-free isolator in facility 4. Total number of mice and collection time points of fecal samples are indicated. **(B)** Relative abundance of fecal microbiota composition at the indicated time points. Abundance of individual strains is shown as relative abundance and expressed as% of cumulative 16S rRNA gene copy numbers of all OMM^12^ strains. One bar corresponds to one mouse. **(C)** PCoA based on the distance matrix of Bray–Curtis dissimilarity of relative OMM^12^ abundance profiles shows the effect of time after inoculation. Points are colored by time (days) after inoculation. Samples taken from two mice are connected to visualize their trajectory during time.

Five European germ-free rodent facilities participated in this study. All mouse experiments were approved by the local authorities and performed according to the legal requirements. Detailed questionnaires were distributed to record the mouse husbandry conditions in the different breeding facilities (Table 1). Germ-free status was routinely confirmed by aerobic and anaerobic culture as well as Sytox green (Invitrogen) and Gram staining (Harleco) of caecal contents to detect unculturable contaminants. C57BL/6J *Agr2*^–/–^ mice were provided by David Erle (Park et al., 2009) and re-derived germ-free from *Agr2*^±^ and *Agr2*^–/–^ conventional mice as described and colonized with OMM^12^ to generate an isobiotic mouse line (Herp et al., 2019). Agr2 encodes a disulfide isomerase that is required for folding and export of the mucin Muc2. For the experiment shown in Figure 1, germ-free *Agr2*^±^ mice were used. *Agr2*^±^ mice behave like wild-type mice in terms of Muc2 secretion and mucus structure (Bergstrom et al., 2014). For all other experiments, wild-type C57BL/6J mice were used.

**TABLE 1:** Description of housing conditions at Germ-free animal facilities.

	**1**	**2**	**3**	**4**	**5**
Housing type	Isolators (TSE Systems, PLEXX)	Isolator (metal + plastic)	Isolator	Gnotocage (Thermo Fischer Scientific, metal + plastic)	Trexler-type plastic isolators
Bedding material	Wood chip (SAFE select)	AsBe-wood GmBH, bedding poplar	Wood shavings	Abedd espe classic; 2,5 mm [LtE E-001]	JELUXYL-SAWI HW 300/500 (JELU-WERK J. Ehrler GmbH & Co., Germany)
Enrichment	Kleenex tissue paper, res plastic nest-houses	Nestlets (Enviro-dri from Bedding Natur), pieces of kitchen paper towels from time to time	Nestlets	Nestlets (Zoonlab, 3097055)	Lignocel nesting small (Velaz, CZ)
Chow provider	Kilba Nafag	Ssniff Spezialdiäten GmbH, Soest, Germany	Ssniff Spezialdiäten GmbH, Soest, Germany	Ssniff Spezialdiäten GmbH, Soest, Germany	Ssniff Spezialdiäten GmbH, Soest, Germany
Chow type	Breeding chow (3807)	Mouse breeding, 10 mm (V1124-027)	Grain-based (19% Protein, 3.3% Fett) (V1534-927)	V1124-300	V1126-000 Mouse breeding, extrudate
Chow treatment	Autoclaved	Gamma-irradiated, 50 kGy	Gamma-irradiated, 50 kGy	Autoclaved	Gamma-irradiated, 25 kGy
Water type	0.22 um filtered tap water then autoclaved	Autoclaved (reverse osmosis)	Tap water	Autoclaved ampuwa; fresenius)	Autoclaved non-chlorinated tap water
Mouse genotype	C57BL/6JZTm	C57BL/6JZtm	C57BL/6J	C57BL/6JZtm	C57BL/6J
Mouse supplier	Clean mouse facility, University of Bern, then bred independently for >10 generations	Mice originally from Jackson Laboratory, delivered as GF colony from Ulm	Own breeding, originally from facility 2	Mice from facility 2	Mice bred in germ-free conditions for >10 generations
Sterility control	Selective culture, Sytox-green quantitative flow cytometry, H2 production			Selective culture, Gram-staining	Selective culture, Gram-staining
Day/night cycle	12:12-h light-dark cycles	12:12-h light-dark cycles	12:12-h light-dark cycles	12:12-h light-dark cycles	12:12-h light-dark cycles
Temperature (°C)	22°C	20–22°C	22°C	20–24°C	22 ± 2°C
Humidity (%)	50–56%	50–55%	56%	45–55%	50–60%

### Bacterial Cultivation

All glassware and media used for cultivation were kept under anoxic conditions (3% H_2_, rest N_2_) in an anaerobic chamber for at least 2 days before the start of the experiment.

Glycerol cryostocks (for preparation see Brugiroux et al., 2016) of individual OMM^12^ strains (Table 2) were thawed in a 1% Virkon S (V.P. Products) solution (37°C) and the entire content of the vial was transferred into 100 ml Wheaton glass serum bottles (Sigma) sealed with a butyl rubber stoppers (Geo-Microbial Technologies) containing 10 ml of Anaerobic Akkermansia Medium (AAM; 18.5 g l^–1^ brain heart infusion (BHI), 5 g l^–1^ yeast extract, 15 g l^–1^ trypticase soy broth, 2.5 g l^–1^ K_2_HPO_4_, 1 mg l^–1^ haemin, 0.5 g l^–1^ glucose, 0.4 g l^–1^ Na_2_CO_3_, 0.5 g l^–1^ cysteine hydrochloride, 5 mg l^–1^ menadione, 3% complement-inactivated fetal calf serum). These subcultures were gassed (7% H_2_, 10% CO_2_, 83% N_2_) and incubated at 37°C for 24 h.

**TABLE 2:** Growth conditions for OMM^12^ strains.

**Strain**	**Cultivation time (days)**
*Clostridium innocuum* I46 DSM 26113	1
*Bacteroides caecimuris* I48 DSM 26085	1
*Lactobacillus reuteri* I49 DSM 32035	1
*Bifidobacterium longum* subsp. *animalis* YL2 DSM 26074	1
*Muribaculum intestinale* YL27 DSM 28989	1
*Flavonifractor plautii* YL31 DSM 26117	1
*Clostridium clostridioforme* YL32 DSM 26114	1
*Akkermansia muciniphila* YL44 DSM 26109	2
*Turicimonas muris* YL45 DSM 26109	2
*Blautia coccoides* YL58 DSM 26115	1
*Acutalibacter muris* KB18 DSM 26090	2
*Enterococcus faecalis* KB1 DSM 32036	1

Subsequently, 100 μl of each subculture was transferred into a 100 ml Wheaton glass serum bottle containing 10 ml of AAM. These cultures were gassed (7% H_2_, 10% CO_2_, 83% N_2_) and incubated at 37°C for 1 or 2 days depending on the growth rate (Table 2). Afterward, culture purity of each strain was confirmed by Gram staining and 16S rRNA gene sequencing. The OD_600_ of individual cultures was determined and all cultures were adjusted to the lowest OD_600_ value by dilution. The respective culture volumes of all strains were transferred into a 50 ml Falcon tube under anoxic conditions. For cryopreservation, glycerol supplemented with palladium black crystals (Sigma-Aldrich) was added to these bacterial mixtures [final concentration of 10% (v/v)]. 1 ml aliquots were prepared in 1.5 ml glass vials (Sigma-Aldrich), sealed with butyl-rubber stoppers (Wheaton) and aluminum crimp seals (Sigma-Aldrich). Mixtures were frozen at −80°C within 1 h of preparation. Frozen aliquots were shipped to the different facilities on dry ice and stored at −80°C.

### Inoculation of Mice and Fecal Sampling

The frozen OMM^12^ mixtures were thawn in a 1% Virkon S (V.P. Produkte) disinfectant solution (37°C) and used for inoculation of germ-free mice in gnotocages or germ-free isolators. In any case, the mixture was used within 30 min after thawing. Mice were inoculated by gavage (50 μl orally, 100 μl rectally). Exposure of the mixture to oxygen was restricted to a short time (up to 5 min). For the double inoculation protocol, inoculation was repeated 72 h after the initial inoculation using the same protocol. To confirm the colonization of the 12 strains, fresh fecal pellets were obtained and frozen at −80°C within 30 min.

### gDNA Extraction From Fecal Samples

Fecal samples were shipped on dry ice. DNA extraction and qPCR were performed centralized at the same laboratory to minimize experimental bias, which is known to be introduced by laboratory-specific experimental procedures. Fecal gDNA was either extracted using the QIAamp DNA Stool Mini Kit (Qiagen; time course shown in Figure 1) or a phenol-chloroform based protocol (all other experiments). The QIAamp DNA Stool Mini Kit protocol was performed following the manufacturer’s instructions with the following modifications. An initial bead-beating step using differentially sized beads [Zirkonia beads: 0.5–0.75 mm (BioSpec products) and acid-washed glass beads: <100 μm (Sigma-Aldrich)] was included and 20 mg/ml lysozyme was added to the lysis buffer. gDNA extraction using the phenol-chloroform based protocol was performed as described previously (Herp et al., 2019). The resulting gDNA was purified using the NucleoSpin gDNA clean-up kit (Macherey-Nagel).

### Quantitative PCR of Bacterial 16S rRNA Genes

Quantitative PCR (qPCR) was performed as described previously (Brugiroux et al., 2016). All samples were analyzed by the same person in a centralized way. Briefly, OMM strain-specific 16S rRNA primers and hydrolysis probes were used for amplification. Standard curves using linearized plasmids containing the 16S rRNA gene sequence of the individual OMM^12^ strains were used for absolute quantification of 16S rRNA gene copy numbers of individual strains. Since the fecal weight was not always available, 16S rRNA gene copy numbers were normalized to equal volumes of extracted DNA, assuming that DNA extraction is equally efficient between different samples. We confirmed that there is a linear relationship between stool weight and extracted DNA concentration (Supplementary Figure S1).

### Statistical Analysis

For comparison of absolute abundance levels of OMM^12^ strains between experiments, Kruskal–Wallis test with Dunn’s multiple comparison test was performed using GraphPad Prism version 5.01 for Windows (GraphPad Software). *p*-values below 0.05 were considered as statistically significant (^∗^*p* < 0.05, ^∗∗^*p* < 0.01, ^∗∗∗^*p* < 0.001).

The vegist function of the R library vegan version 2.5–4 was employed to obtain Bray–Curtis (BC) dissimilarities between the samples based on relative abundance estimates. Principal coordinate analysis was performed in R using ade4 package and figures were generated using the ggplot2 library. Permutational multivariate analyses of variance (PERMANOVA) were performed in R using the function adonis. We used the function capscale with the variable “facility” as constraint to estimate the effect of the facility on the overall BC variance. Statistical significance of the ordinations as well as confidence intervals for the variance were determined by an ANOVA-like permutation test (functions permutest and anova.cca) with 5,000 permutations. The heatmap (Supplementary Figure S1) was generated using the ComplexHeatmap library on the BC values.

To estimate the within facility stability we calculated the mean of all pairwise BC dissimilarities of community profiles of mice from the same facility. Between-facility estimates were calculated using the mean value of all pairwise distances between the community profile of each mouse of one facility to the community profile of all mice of different facilities. R scripts are available under https://github.com/philippmuench/OligoMM-facilities.

## Results

### OMM^12^ Colonization Dynamics After Oral Inoculation

**FIGURE 2:**
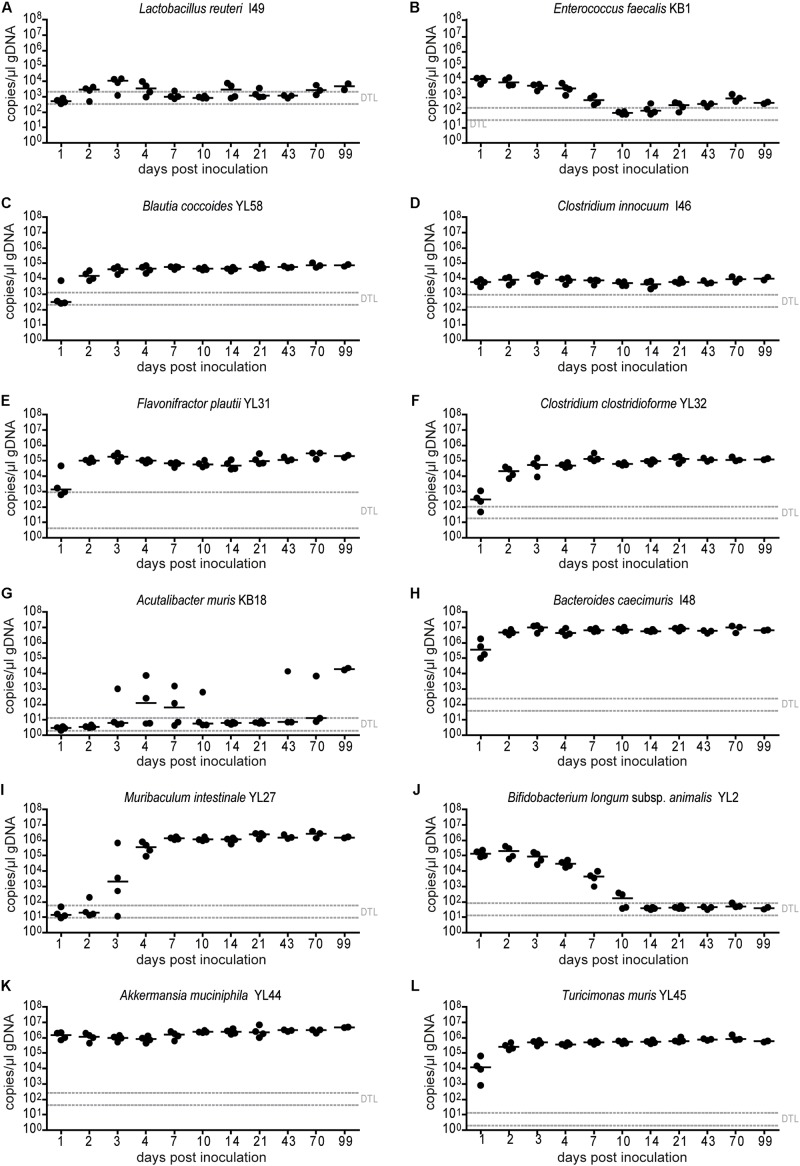
Absolute abundance of individual OMM^12^ strains in time course analysis of OMM^12^ colonization in germ-free mice. Germ-free mice were inoculated with the OMM^12^ mixture and kept in a germ-free isolator. Fecal samples were collected at different time points for microbiota analysis. Absolute abundance of each strain was determined by a strain-specific qPCR assay and is plotted as 16S rRNA gene copy numbers of the individual strains per μl of extracted gDNA: **(A)** *Lactobacillus reuteri* I49, **(B)** *Enterococcus faecalis* KB1, **(C)** *Blautia coccoides* YL58, **(D)** *Clostridium innocuum* I46, **(E)** *Flavonifractor plautii* YL31, **(F)** *Clostridium clostridioforme* YL32, **(G)** *Acutalibacter muris* KB18, **(H)** *Bacteroides caecimuris* I48, **(I)** *Muribaculum intestinale* YL27, **(J)** *Bifidobacterium longum* subsp. *animalis* YL2, **(K)** *Akkermansia muciniphila* YL44, **(L)** *Turicimonas muris* YL45. Statistical analysis was performed using Kruskal-Wallis test with Dunn’s multiple comparison test (^∗^*p* < 0.05, ^∗∗^*p* < 0.01, ^∗∗∗^*p* < 0.001). Green symbols indicate samples collected <20 days post inoculation. Dotted lines indicate detection limits.

In order to determine the time necessary for stable community formation, we tested temporal dynamics of the OMM^12^ community after oral inoculation to germ-free mice in one facility. Changes in OMM^12^ microbial community composition were closely monitored over time in fecal samples for 99 days (Figure 1A). In this experiment, we used two germ-free breeders from a heterozygous C57BL/6J *Agr2*^±^ breeding, which were reconstituted with the OMM^12^ consortium to generate an isobiotic mouse line (Herp et al., 2019). Fecal community composition, represented as relative abundance profiles quantified by strain-specific qPCR, rapidly changed within the first week after OMM^12^ inoculation. Beyond day 7 post inoculation, OMM^12^ community stabilized and profiles remained highly similar afterward (Figure 1B). Principal Coordinates Analysis (PCoA) of Bray–Curtis dissimilarities showed a gradual shift with time toward a more similar community (Figure 1C). At early time points, high abundance of *Bifidobacterium longum* subsp. *animalis* YL2 and *Enterococcus faecalis* KB1 was observed, which gradually declined within the first week (Figures 2B,J). Inverse colonization dynamics were seen for several other strains (Figure 2). Notably, *Muribaculum intestinale* YL27 took 4 days to reach detectable levels in all mice (Figure 2I). Colonization levels for *Clostridium innocuum* I46 and *Akkermansia muciniphila* YL44 were constant throughout (Figure 2K). The number of 16S rRNA gene copies of *Acutalibacter muris* KB18 remained below the detection limit in the majority of fecal samples, which was observed previously in stably colonized OMM^12^ mice (Brugiroux et al., 2016). We conclude that the OMM^12^ consortium adopts a stable composition between 10 and 20 days post-inoculation. Therefore, we reasoned, that a 3-week colonization phase after inoculation is sufficient to verify successful colonization of OMM^12^ in the feasibility trial outlined below.

### A Single Inoculation Does Not Lead to Reproducible Introduction of *Muribaculum intestinale* in Different Germ-Free Mouse Facilities

**FIGURE 3:**
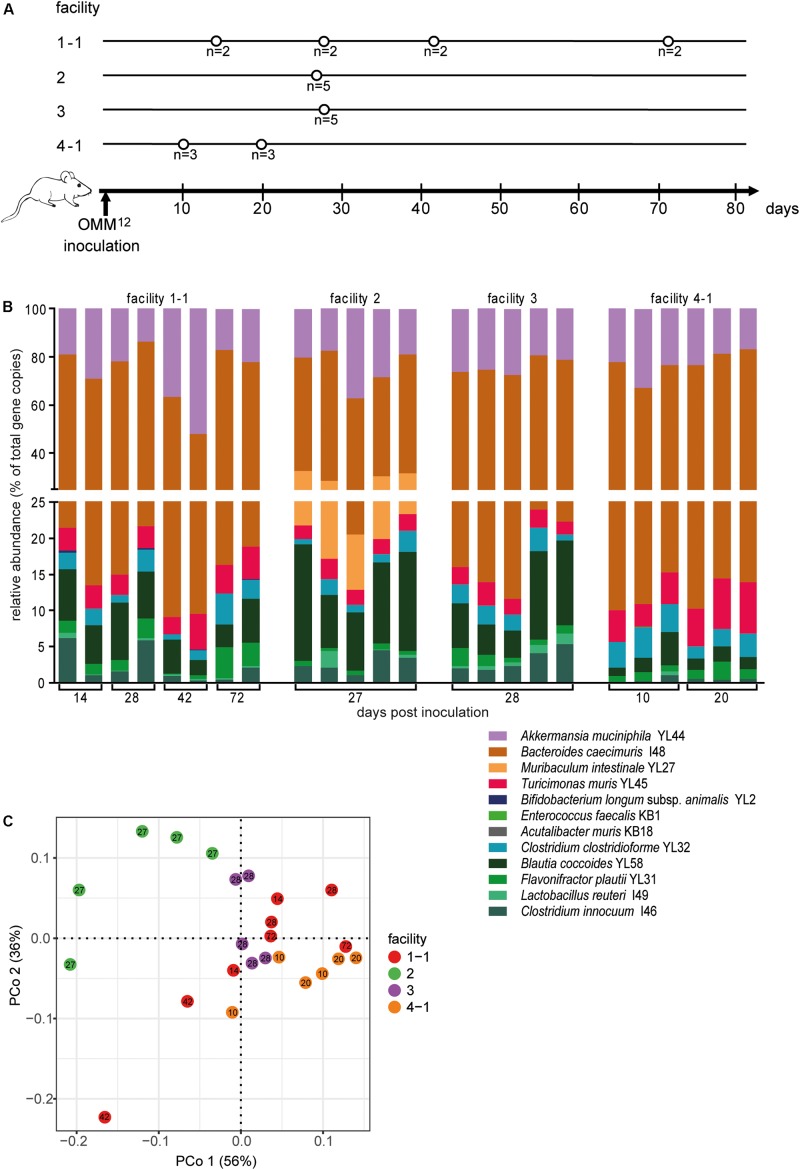
Colonization dynamics of OMM^12^ mice in four different germ-free facilities after single-dose inoculation. **(A)** Experimental scheme. Germ-free C57BL/6J mice were inoculated with the OMM^12^ mixtures and kept in germ-free isolators or gnotocages at four different animal facilities (1-1, 2, 3, 4-1); the number of mice and collection time points of fecal samples are indicated. **(B)** Fecal microbiota composition at the different time points, displayed as relative abundance and expressed as the fraction of cumulated 16S rRNA gene copy numbers. One bar corresponds to one mouse. **(C)** PCoA based on Bray–Curtis dissimilarity (relative abundances) between samples obtained from mice in different facilities. Points are colored by facility.

Next, we investigated, whether germ-free mice can be reproducibly associated with OMM^12^ in different germ-free mouse facilities across four participating institutions in Germany and Switzerland. Germ-free C57Bl/6J mice (*n* = 2–5) at different facilities were orally inoculated once with the same batch of inoculum, to avoid variations introduced by differences in the inoculum. We obtained feces from the animals and different time points (day 10–72) post-inoculation (Figure 3A). Facility-specific characteristics are outlined in Table 1.

Relative OMM^12^ abundance profiles of mice from different facilities showed, at large, high similarity (Figure 3B). PCoA based on Bray–Curtis dissimilarity (relative abundances) between samples showed that community composition was overall similar with the exception of facility 2, which clustered separately (Figure 3C). We used canonical analysis of principal coordinates (CAP; Anderson and Willis, 2003) to estimate the influence of the facility on the beta diversity. CAP analysis constrained by the facility revealed that the facility explains 35% of the overall variance of Bray–Curtis dissimilarity between samples from different facilities (*p* < 0.001). Based on PERMANOVA analysis of Bray–Curtis dissimilarities, facility “2” was clearly distinguishable from the other three facilities (Figure 3C and Supplementary Table S1).

**FIGURE 4:**
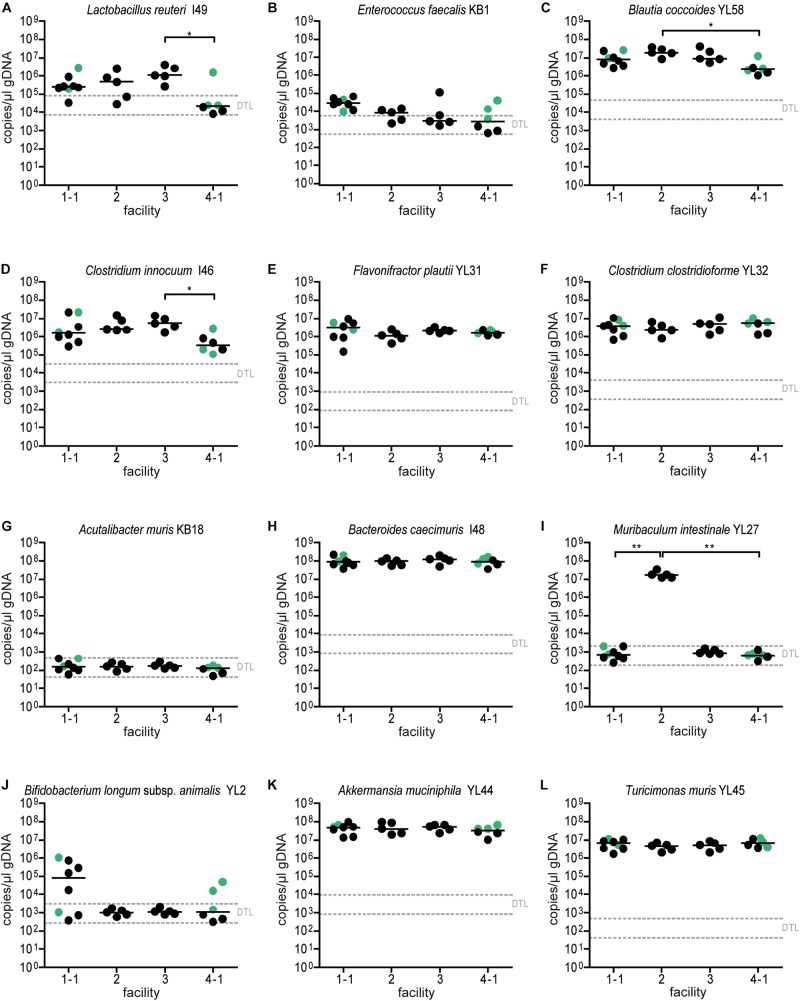
Colonization dynamics of OMM^12^ mice in four different germ-free facilities after single-dose inoculation: absolute abundance of individual OMM^12^ strains. Absolute abundance of each strain was determined using a strain-specific qPCR assay for the experiment described in Figure 3, Data are plotted as 16S rRNA gene copy numbers of the individual strains per μl of extracted gDNA: **(A)** *Lactobacillus reuteri* I49, **(B)** *Enterococcus faecalis* KB1, **(C)** *Blautia coccoides* YL58, **(D)** *Clostridium innocuum* I46, **(E)** *Flavonifractor plautii* YL31, **(F)** *Clostridium clostridioforme* YL32, **(G)** *Acutalibacter muris* KB18, **(H)** *Bacteroides caecimuris* I48, **(I)** *Muribaculum intestinale* YL27, **(J)** *Bifidobacterium longum* subsp. *animalis* YL2, **(K)** *Akkermansia muciniphila* YL44, **(L)** *Turicimonas muris* YL45. Statistical analysis was performed using Kruskal-Wallis test with Dunn’s multiple comparison test (^∗^*p* < 0.05, ^∗∗^*p* < 0.01, ^∗∗∗^*p* < 0.001). Green points indicate samples collected <20 days post inoculation.

Absolute abundance, as determined by qPCR revealed that 7 of the 12 species were detected in all mice at the different facilities at comparable levels (Figure 4). The absolute abundance of *Lactobacillus reuteri* I49, *E. faecalis* KB1, *B. longum* subsp. *animalis* YL2 and *A. muris* KB18 varied substantially between the facilities and was below the detection limit in most samples (Figures 4A,B,G,J). Additionally, *Muribaculum intestinale* YL27 was only detectable in facility “2” (Figure 4I). This may explain the notable different community profile of mice from this facility (Figure 3C and Supplementary Table S1). We reasoned that single inoculation of mice might not be sufficient to ensure reliable colonization of *M. intestinale* YL27.

### Double-Dose Application Increases Reproducibility of OMM^12^ Colonization in Different Germ-Free Mouse Facilities

**FIGURE 5:**
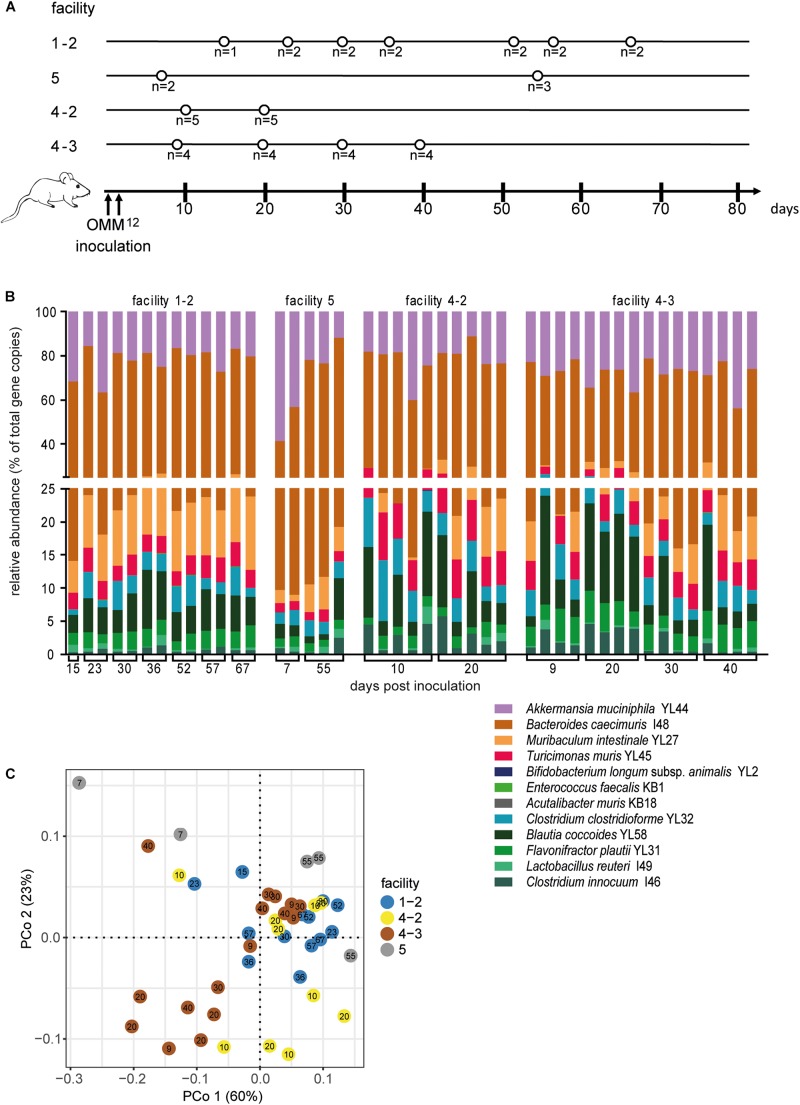
Colonization dynamics of OMM^12^ mice in four different germ-free facilities after double-dose inoculation reveals high reproducibility. **(A)** Experimental scheme. Germ-free C57BL/6 mice were inoculated twice with the OMM^12^ mixtures and kept in germ-free isolators or gnotocages at different facilities (1-2, 5, 4-2, 4-3). In case of facility “1” and “4,” inoculations were done on several independent occasions. The number of mice and collection time points of fecal samples are indicated. **(B)** Fecal microbiota composition at the different time points. Microbiota composition is shown as relative abundance and expressed as the fraction of cumulated 16S rRNA gene copy numbers. One bar corresponds to one mouse. **(C)** PCoA based on the distance matrix of Bray–Curtis dissimilarity of relative OMM^12^ abundance profiles shows samples obtained from mice in different facilities. Points are colored by facility.

**FIGURE 6:**
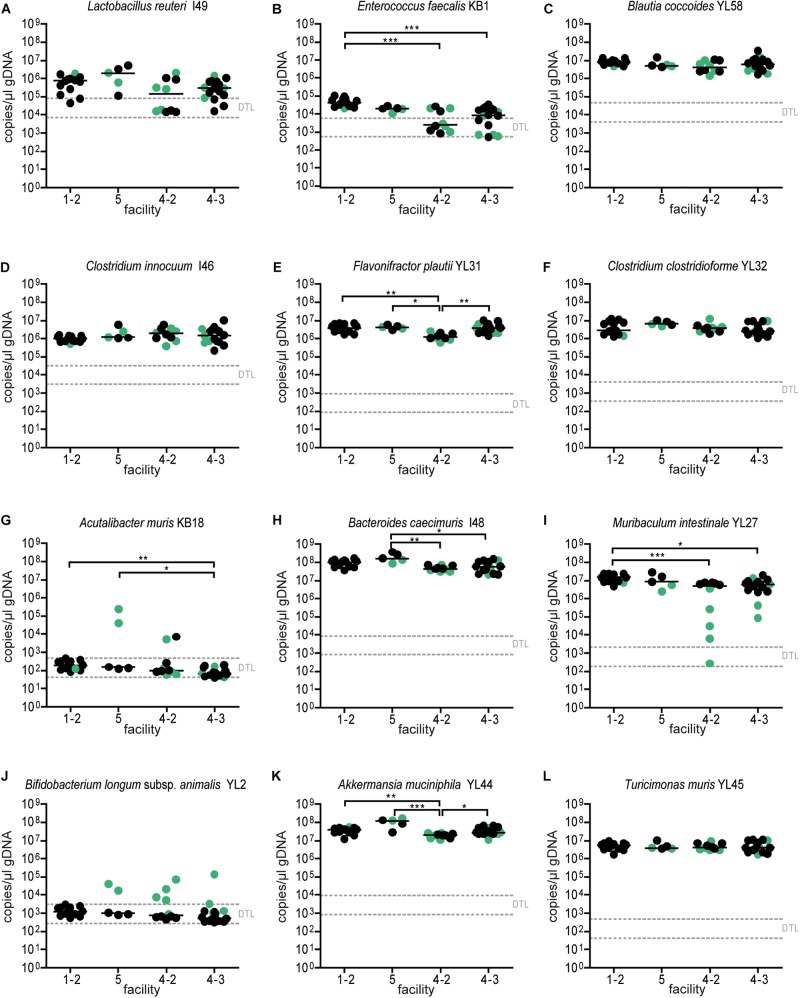
Absolute abundance of OMM^12^ strains in different germ-free facilities after double-dose inoculation. Absolute abundance of each strain was determined using a strain-specific qPCR assay for the experiment described in Figure 5, Data are plotted as 16S rRNA gene copy numbers of the individual strains per μl of extracted gDNA: **(A)** *Lactobacillus reuteri* I49, **(B)** *Enterococcus faecalis* KB1, **(C)** *Blautia coccoides* YL58, **(D)** *Clostridium innocuum* I46, **(E)** *Flavonifractor plautii* YL31, **(F)** *Clostridium clostridioforme* YL32, **(G)** *Acutalibacter muris* KB18, **(H)** *Bacteroides caecimuris* I48, **(I)** *Muribaculum intestinale* YL27, **(J)** *Bifidobacterium longum* subsp. *animalis* YL2, **(K)** *Akkermansia muciniphila* YL44, **(L)** *Turicimonas muris* YL45. Statistical analysis was performed using Kruskal–Wallis test with Dunn’s multiple comparison test (^∗^*p* < 0.05, ^∗∗^*p* < 0.01, ^∗∗∗^*p* < 0.001). Green color indicate samples collected <20 days post inoculation.

Since single application of the OMM^12^ mixture did not lead to reproducible colonization of OMM^12^ strains across different germ-free mouse facilities, we aimed to amend the protocol. Previous work indicated that consecutive inoculations might increase the chance of successful introduction of oxygen-sensitive members of a consortium (Becker et al., 2011) (Taconic protocols). Therefore, we modified the initial inoculation protocol and applied the mixture twice with 72 h in-between inoculations. This time, three facilities participated and two independent trials were performed at facility “4.” We found that relative OMM^12^ abundance profiles of mice from different facilities and trials were rather uniform (Figure 5B). PCoA based on Bray–Curtis dissimilarity (relative abundances) between samples showed that community composition was overall similar between facilities (Figure 5C). CAP analysis constrained by the facility revealed that in this trial, the facility explains 20% of the overall variance in Bray–Curtis dissimilarity of the data (*p* < 0.001). In this trial, no obvious clustering was apparent between samples from different facilities (Figure 5C), yet based on PERMANOVA analysis of Bray–Curtis dissimilarities, some differences between facilities were observed (Supplementary Table S2). *Muribaculum intestinale* YL27 was reliably detected in all mice colonized in the different facilities (Figure 6I), which is a substantial improvement compared to the single-dose experiment. However, levels of *Lactobacillus reuteri* I49, *E. faecalis* KB1, *B. longum* subsp. *animalis* YL2 and *A. muris* KB18 still varied between the tested facilities and were below detection limit in some samples (Figure 6).

**FIGURE 7:**
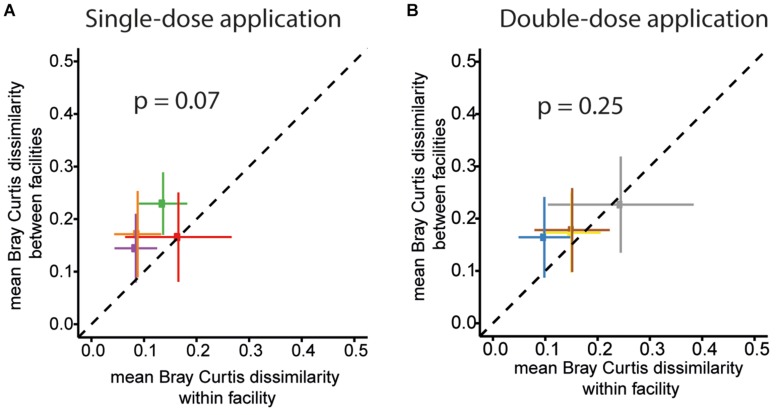
OMM^12^ community profiles within and between different facilities for the two trials. The mean of pairwise BC dissimilarity values of the community profiles of mice housed in the same facility (within facility analysis) was plotted against the mean pairwise BC dissimilarity values of the community profile of mice located in different facilities for single-dose inoculation trial for **(A)** Single-dose inoculation and **(B)** double-dose inoculation trial. For single-dose inoculation, the mean BC dissimilarity is 0.12 ± 0.04 (mean ± SD) and 0.18 ± 0.04, within and between the facilities, respectively (*p*-value = 0.07, paired two-sided *t*-test). For double-dose inoculation, the mean BC dissimilarity is 0.16 ± 0.06 and 0.19 ± 0.03 for within and between the facilities, respectively (*p*-value = 0.25; paired two-sided *t*-test).

Finally, we assessed variations in overall community profiles within and between the facilities for the two different trials. To this end, we compared Bray–Curtis dissimilarities between samples of the same facility to dissimilarities between samples of different facilities. An overview of the pair-wise compositional Bray–Curtis dissimilarity of relative community profiles of mice between and across facilities and studies (single-dose and double-dose) is shown in Supplementary Figure S2. For the first trial (single-dose inoculation), the mean of pairwise Bray–Curtis dissimilarity values of the community profile of mice housed in the same facility is lower than the mean pairwise Bray–Curtis dissimilarity between samples from mice located in different facilities. The mean Bray–Curtis dissimilarity for within-and between facility are 0.12 ± 0.04 (mean ± SD) and 0.18 ± 0.04, respectively; *p*-value = 0.07; paired two-sided *t*-test (Figure 7A). For the double-dose trial, the mean Bray–Curtis dissimilarity was lower (0.16 ± 0.06 and 0.19 ± 0.03 for within and between facilities, respectively; *p*-value = 0.25; paired two-sided *t*-test, Figure 7B).

## Discussion

Variations in the gut microbiota within and between animal facilities can be a major factor accounting for the lack of reproducibility of animal models of human biology and disease (Franklin and Ericsson, 2017). Several guidelines were established to optimally control for microbiota differences when using different genetic strains of experimental mice within an animal unit (McCoy et al., 2017; Wullaert et al., 2018). Gnotobiotic mouse models based synthetic microbial consortia are becoming increasingly popular. In particular, these models offer the opportunity to generate “isobiotic” mice, which may significantly enhance experimental reproducibility across different institutions (Macpherson and McCoy, 2015).

This study reports on the first comparative inter-facility trial conducted to evaluate and optimize the effectiveness of a protocol for colonization of germ-free mice with a synthetic bacterial community. The majority of current protocols for introducing synthetic bacterial communities to germ-free mice use mixtures generated from fresh pure bacterial cultures for inoculations (Faith et al., 2011; Desai et al., 2016; Gomes-Neto et al., 2017). These protocols require a sophisticated cultivation setup, including devices for anaerobic bacterial cultivation. Our protocol overcomes these limitations by generating frozen aliquots of a mixture of the strains, which can be distributed, thawed and directly applied. We note that in our trial, frozen aliquots of bacterial mixtures remain viable for at least 18 months at −80°C. Another common method for generating gnotobiotic mice is by co-housing with a colonized donor animal (Geuking et al., 2011), a setup which requires no expertise for bacterial cultivation. When using this approach, it should be considered that serial passage of a bacterial community within the same facility or in between facilities, e.g., through breeding, might promote genomic diversification of the individual community members by consecutive rounds of within-host selection (Robinson et al., 2018). The evolved bacterial community would differentiate genetically and functionally from the parental strains over time. To date, the degree and temporal course of within-host evolution of microbial communities is not known and experimental data are only available for evolution of individual bacterial populations in the gut (Leatham et al., 2005; De Paepe et al., 2011; Barroso-Batista et al., 2014). Based on the analysis of individual bacterial populations, it is expected that mutants emerge rapidly and are selected based on improved competitiveness within days. In order to retain genetic identity of a minimal bacterial consortium, it is advisable to regenerate gnotobiotic mouse lines every 18–24 months using original cultures. This period is the result of cost-benefit considerations, keeping the degree of genomic diversification within acceptable boundaries but at the same time, minimizing costs and experimental efforts.

In this trial, we used aliquots of the same batch of frozen OMM^12^ mixed cultures for the inoculations. This allowed us to compare efficiency of inoculation between different facilities using the same starting material. Thereby the inoculum could be eliminated as confounding factor in microbial community establishment in the gut. The gut microbiome of mice can be significantly influenced by husbandry-related factors, such as type of laboratory animal diet (Hildebrandt et al., 2009; Ooi et al., 2014) water (Sofi et al., 2014), housing effects, genetic background (Deloris Alexander et al., 2006; Hildebrand et al., 2013) and a wide range of other environmental and stress-related factors (Bangsgaard Bendtsen et al., 2012). Many of these variables are likely to differ across germ-free animal facilities and account for the small but measurable differences in OMM^12^ community composition observed in our study. Further, due to facility-specific differences in procedures and experimental protocols, it was not possible to match sex, age and number of inoculated mice and obtain fecal samples at matched time points post inoculation. This may account for part of the facility-dependent differences and should be optimized in future trials.

Our results suggest that a double-dose application with a 72 h interval improves the engraftment of *Muribaculum intestinale* YL27. Representatives of the Muribaculaceae family (former S24-7) are highly diverse and dominant members of the murine gut microbiota (Lagkouvardos et al., 2019). *M. intestinale* YL27, the first cultured representative, is strictly anaerobic and genome-based prediction indicates the potential to degrade complex carbohydrates (Lagkouvardos et al., 2016; Lee et al., 2019). In our study, *M. intestinale* YL27 showed slow colonization dynamics after oral inoculation compared to all other OMM^12^ strains disclose that expansion time can take up to 20 days. The oxidation/reduction (O/R) potential is increased in germ-free mice (+200 mV) compared to conventional mice but becomes reduced in response to colonization with a complex microbiota (−200 mV) (Celesk et al., 1976). As *M. intestinale* YL27 is oxygen-sensitive, we reason that high O/R potential in the germ-free mouse gut may inhibit its expansion early after inoculation. Although a recent study showed that the luminal contents of germ-free mice can chemically consume oxygen (e.g., via lipid oxidation reactions), the gut lumen of germ-free and antibiotic-treated mice may also exhibit increased luminal oxygen concentration compared to mice colonized with a complex microbiota (Friedman et al., 2018). Oxygen-tolerant members of the microbiota are among the first colonizers in a germ-free environment, after antibiotic treatment or in the course of intestinal colonization of the neonatal gut. They are thought to consume oxygen and anaerobic electron acceptors and to reduce the O/R potential sufficiently for oxygen-sensitive strains to colonize (Reese et al., 2018). Presumably, colonization dynamics of the OMM^12^ consortium are subject to similar principles: Some members of the consortium (*Enterococcus faecalis, Bifidobacterium longum*) that are also among the early colonizers of the human neonatal gut predominate at early colonization stages. Obligate anaerobic bacteria (*Bacteroides acidifaciens*, Clostridiales) follow with a delay of 3 days. We assume that a second dose of OMM^12^ administered when the O/R potential has already been lowered by the initial colonizers increases the chance of successful engraftment of obligate anaerobes such as *Muribaculum intestinale*.

In summary, our study demonstrates that germ-free mice in different facilities can be reproducibly associated with the OMM^12^ synthetic bacterial community, employing a protocol using previously frozen aliquots of a mixture of the strains. Furthermore, application of the consortium at two consecutive time points increased the chance of community engraftment. We envision that guidelines and validated protocols for generation of gnotobiotic models based on synthetic microbial communities will contribute to optimizing experimental reproducibility in this intense area of research.

## Data Availability Statement

All datasets generated for this study are included in the article/Supplementary Material.

## Ethics Statement

The animal study was reviewed and approved by the Regierung von Oberbayern, Kantonales Veterinäramt Zürich, and the Lower Saxony State Office for Consumer Protection and Food Safety (LAVES).

## Author Contributions

BS and CE conceived and designed the experiments. CE, DR, PM, MBe, MBa, ES, MS, DS, and AL performed the experiments. CE and PM analyzed the data. JF, AB, and BS contributed the materials and analysis tools. BS coordinated the project and wrote the original draft. All authors reviewed and edited the draft of the manuscript. Correspondence and requests for materials should be addressed to BS.

## Conflict of Interest

The authors declare that the research was conducted in the absence of any commercial or financial relationships that could be construed as a potential conflict of interest.

## Footnotes

1 https://www.dsmz.de/
