# Supplementary material for: Reproducible Colonization of Germ-Free Mice With the Oligo-Mouse-Microbiota in Different Animal Facilities
Source: Front Microbiol. 2020 Jan 10;10:2999. doi: 10.3389/fmicb.2019.02999 (PMC6965490; doi:10.3389/fmicb.2019.02999)
Supplement: Supplementary file 1 [file Data_Sheet_1.DOCX]

**Supplementary Material**

**Colonization of germ-free mice with the Oligo-Mouse-Microbiota is reproducible in different animal facilities**

**Claudia Eberl^1^, Diana Ring^1,2^, Philipp C. Münch^1,3^, Markus Beutler^1^, Marijana Basic^4^, Emma Caroline Slack^5^, Martin Schwarzer^6^, Dagmar Srutkova^6^, Anna Lange^7^, Julia S. Frick^7^, André Bleich^4^ and Bärbel Stecher^1,2#^**

^1^Max-von-Pettenkofer Institute, LMU Munich, 80377 Munich, Germany

^2^German Center for Infection Research (DZIF), partner site LMU Munich, Munich, Germany

^3^Department for Computational Biology of Infection Research, Helmholtz Center for Infection Research, Brunswick, Germany

^4^Institute for Laboratory Animal Science and Central Animal Facility, Hannover Medical School, 30625 Hannover, Germany

^5^Institute of Food, Nutrition and Health, ETH Zurich, 8093 Zurich, Switzerland

^6^Institute of Microbiology of the Czech Academy of Sciences, 54922 Novy Hradek, Czech Republic

^7^Institute of Medical Microbiology and Hygiene, University of Tübingen, Tübingen, Germany

^8^German Center for Infection Research (DZIF), partner site Tübingen, Tübingen, Germany

^#^corresponding author

**Supplementary Figure Legends**

**Figure S1: Linear correlation of fecal weight and extracted gDNA concentration**

Fecal pellets from OMM^12^ mice were homogenized and divided in fractions of different weight. gDNA was extracted (see materials and methods) and nucleic acid concentration was determined. A linear relationship was found between fecal weight and extracted gDNA concentration (Pearson correlation coefficient r: 0.9741; p<0.0001).

**Figure S2: Overview of the compositional dissimilarity (BC) of relative community profiles of mice between and across facilities and studies (single-dose and double-dose).**

Shown are pairwise BC dissimilarity values from 0 (blue = two samples have the same composition) to 0.4. Samples are clustered within each facility and within each study using single-linkage. Clustering between facility is based on the mean value of each facility. Mice show higher similarity within the same facility (except two mice that show no similarity to any other sample).

**Supplementary Tables**

**Supplementary Table S1. PERMANOVA analysis of Bray-Curtis dissimilarities of data shown in Figure 3C**

**Supplementary Table S2. PERMANOVA analysis of Bray-Curtis dissimilarities of data shown in Figure 5C**
