# Supplementary figures and images for: Reproducible Colonization of Germ-Free Mice With the Oligo-Mouse-Microbiota in Different Animal Facilities
Source: Front Microbiol. 2020 Jan 10;10:2999. doi: 10.3389/fmicb.2019.02999 (PMC6965490; doi:10.3389/fmicb.2019.02999)

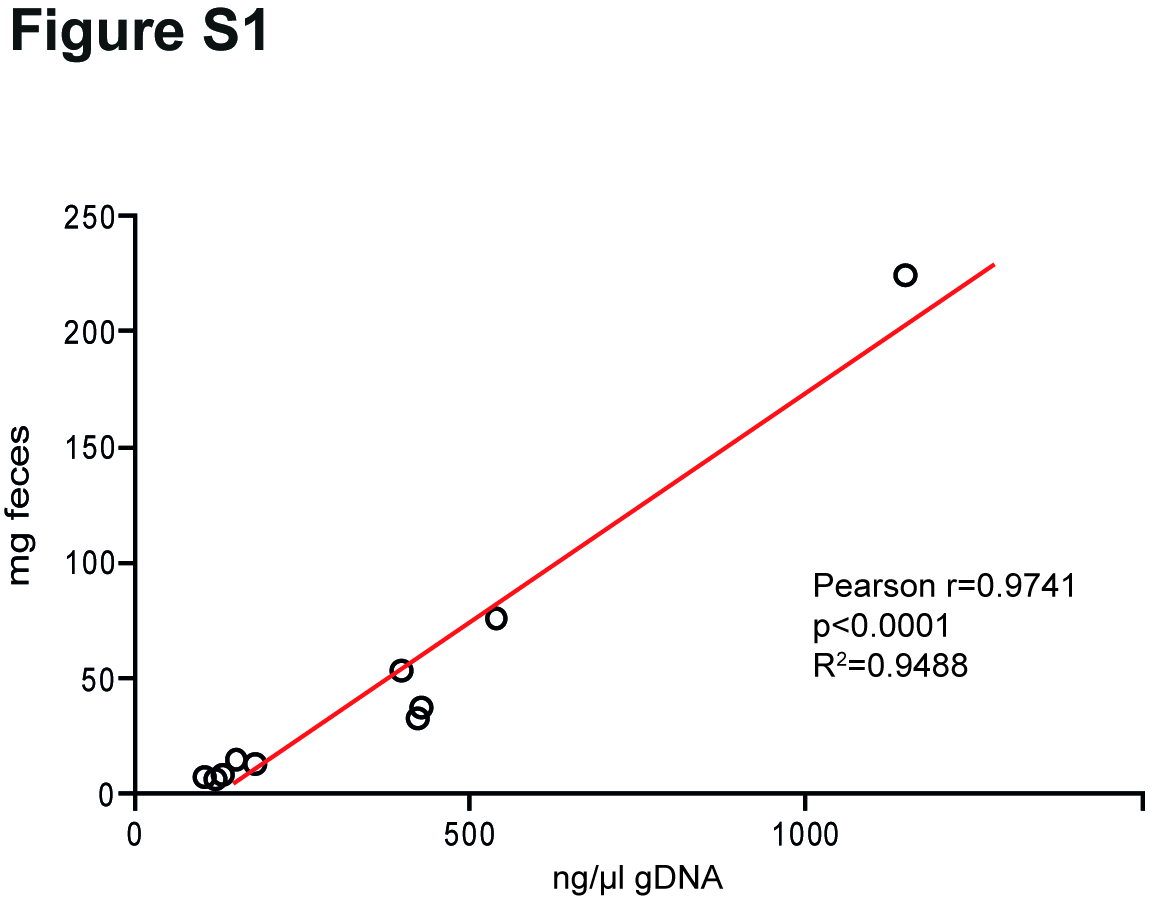

Supplement: Supplementary file 2 [file Image_1.TIF]

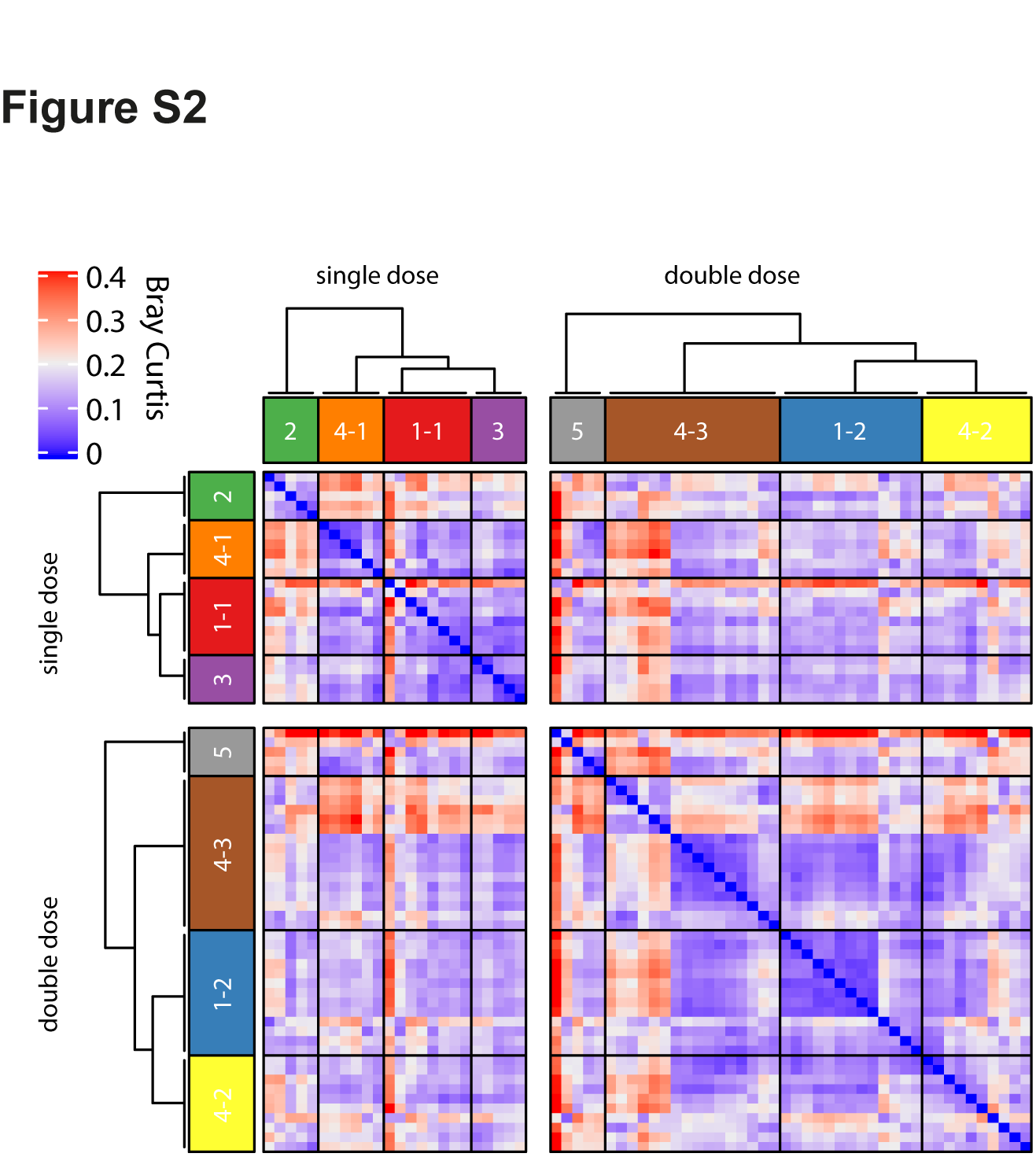

Supplement: Supplementary file 3 [file Image_2.TIF]
